# Modeling the impact of child vaccination (5–11 y) on overall COVID-19 related hospitalizations and mortality in a context of omicron variant predominance and different vaccination coverage paces in Brazil

**DOI:** 10.1016/j.lana.2022.100396

**Published:** 2022-11-17

**Authors:** Gabriel Cardozo Müller, Leonardo Souto Ferreira, Felipe Ernesto Mesias Campos, Marcelo Eduardo Borges, Gabriel Berg de Almeida, Silas Poloni, Lorena Mendes Simon, Ângela Maria Bagattini, Michelle Quarti, José Alexandre Felizola Diniz Filho, Roberto André Kraenkel, Renato Mendes Coutinho, Suzi Alves Camey, Ricardo de Souza Kuchenbecker, Cristiana Maria Toscano

**Affiliations:** a Programa de Pós-graduação em Epidemiologia, Faculdade de Medicina, Universidade Federal do Rio Grande do Sul, Porto Alegre, RS, Brazil; b Observatório Covid-19, Brazil; c Instituto de Física Teórica, Universidade Estadual Paulista, São Paulo, SP, Brazil; d Programa de Pós-Graduação em Ecologia, Instituto de Biociências, Universidade de São Paulo, São Paulo, SP, Brazil; e Departamento de Infectologia, Faculdade de Medicina de Botucatu, Universidade Estadual Paulista, São Paulo, Brazil; f Departamento de Ecologia, Instituto de Ciências Biológicas, Universidade Federal de Goiás, Goiânia, GO, Brazil; g Departamento de Saúde Coletiva, Instituto de Patologia Tropical e Saúde Pública, Universidade Federal de Goiás, Goiânia, GO, Brazil; h Centro de Matemática, Computação e Cognição, Universidade Federal do ABC, Santo André, SP, Brazil; i Instituto de Matemática e Estatística, Departamento de Estatística, Universidade Federal do Rio Grande do Sul, Porto Alegre, RS, Brazil; j Hospital de Clínicas de Porto Alegre, Porto Alegre, RS, Brazil

**Keywords:** COVID-19 vaccines, Vaccination, Infectious disease modeling, Children, SARS-CoV-2 variants

## Abstract

**Background:**

Developing countries have experienced significant COVID-19 disease burden. With the emergence of new variants, particularly omicron, the disease burden in children has increased. When the first COVID-19 vaccine was approved for use in children aged 5–11 years of age, very few countries recommended vaccination due to limited risk-benefit evidence for vaccination of this population. In Brazil, ranking second in the global COVID-19 death toll, the childhood COVID-19 disease burden increased significantly in early 2022. This prompted a risk-benefit assessment of the introduction and scaling-up of COVID-19 vaccination of children.

**Methods:**

To estimate the potential impact of vaccinating children aged 5–11 years with mRNA-based COVID-19 vaccine in the context of omicron dominance, we developed a discrete-time SEIR-like model stratified in age groups, considering a three-month time horizon. We considered three scenarios: No vaccination, slow, and maximum vaccination paces. In each scenario, we estimated the potential reduction in total COVID-19 cases, hospitalizations, deaths, hospitalization costs, and potential years of life lost, considering the absence of vaccination as the base-case scenario.

**Findings:**

We estimated that vaccinating at a maximum pace could prevent, between mid-January and April 2022, about 26,000 COVID-19 hospitalizations, and 4200 deaths in all age groups; of which 5400 hospitalizations and 410 deaths in children aged 5–11 years. Continuing vaccination at a slow/current pace would prevent 1450 deaths and 9700 COVID-19 hospitalizations in all age groups in this same time period; of which 180 deaths and 2390 hospitalizations in children only.

**Interpretation:**

Maximum vaccination of children results in a significant reduction of COVID-19 hospitalizations and deaths and should be enforced in developing countries with significant disease incidence in children.

**Funding:**

This manuscript was funded by the Brazilian Council for Scientific and Technology Development (10.13039/501100003593CNPq – Process # 402834/2020-8).

**Research in context**

**Evidence before this study**

From Nov, 2021 to Mar, 2022 we continuously searched in Pubmed, MedRxiv and WHO weekly report, to find vaccine estimates on impact of vaccinating children aged 5–11 years with mRNA based COVID-19 vaccine in the context of Omicron VOC dominance (speccharacterized by a higher immune evasion, as shown in several works presenting lower vaccines efficacy against this VOC), and found even some studies presenting efficacy on this age group, few of them presented measures of impact of an national based strategy in child vaccination to prevent increasing hospitalizations and deaths related to COVID-19. Also data on developing countries are more difficult to find, what may fuel discussion on the need of risk-benefit studies when adopting this strategy, especially when decision makers and international societies have taken a longer time to recommend it, as recently Joint Commission for Vaccination and Immunization changed its statement to recommend child vaccination on early 2022, after an increase of new cases by introduction of Omicron VOC.

**Added value of this study**

Using a discrete time SEIR-like stratified per age group model, we present data on potential deaths and hospitalizations averted by two strategies of vaccination for children, considering the actual pace and an optimal pace of administration, based on previous campaigns of Brazilian Ministry of Health. Model parameters were determined using best evidence available on vaccine efficacy, also considering that all population was susceptible to infection by Omicron VOC in a 90-day time horizon, between Jan, 2022 and Apr, 2022. As resultant, a significant number of deaths and hospitalizations could be averted, even in actual/slow pace, also averting thousands of potential years of life lost and on millions related to hospital admissions. Also, in an optimal pace, this impact could be at least three times greater, even in sensitivity analysis with lower vaccine efficacies.

**Implications of all the available evidence**

It is well known that vaccines are the main strategy to control course of epidemics and to prevent outbreaks by new Sars-CoV-2 variants. In summary, combining the previous evidences and our data, strengthening national based campaigns for vaccination in an adequate pace is an important step to avert deaths and to reduce potential complications related to long-term COVID-19, especially when incorporating children to increase coverage and to promote an overall protective effect.

## Introduction

COVID-19 had a great impact on developing countries, especially Brazil, which holds the second-largest number of COVID-19-related deaths worldwide.^1^ Due to its large territorial extension, Brazil's COVID-19 epidemic was characterized by regional epidemic curves after the introduction of new variants of concern (VOC), culminating with a large nationwide synchronized wave caused by gamma VOC in mid-2021.^1^ In December 2021 the omicron variant was detected in Brazil, leading to an exponential increase in infections and causing a very high number of cases in all age groups as of early 2022.^2^

The omicron variant is characterized by a large number of mutations in the spike protein, which explains its high capability of transmission and reinfection.^2^ Moreover, these mutations have conferred the capacity to partially escape from immune response.^3^ omicron has a higher reinfection rate compared to the Delta variant, being also capable of infecting vaccinated individuals and causing disease in those partially vaccinated.^2^^,^^4^ As a result, considering that a third (booster) vaccine dose can significantly lower these rates, booster vaccination has been recommended to further protect against omicron.

There is even greater concern about the impact of omicron on children. For children aged between 5 and 11 years, 9562 hospitalizations and 447 deaths by COVID-19 have been registered in Brazil since the beginning of the pandemic until October 17, 2022.^5^^,^^6^ Children can be considered natural reservoirs of SARS-CoV-2 and its variants,^7^ usually presenting mild or asymptomatic disease. Nevertheless, a higher transmission rate of omicron infections among children has been documented, resulting in a sharp increase in COVID-19 hospitalizations in this age group in many countries. In the United States^8^ and in many Brazilian states,^9^ this increase has resulted in a more significant disease burden in children than in any other previous moments of the pandemic.

Vaccination against COVID-19 in Brazil initiated in January 2021 targeting priority groups including elderly and healthcare professionals, and was later extended for all adults. Vaccination of teenagers aged 12–17 years began in September 2021. More recently, in December 2021, results of randomized clinical trials evaluating the efficacy of the vaccine against COVID-19 in children 5–11 years old demonstrated its efficacy and safety, estimating an effectiveness greater than 90% in reducing hospitalizations and deaths by COVID-19 in children.^10^^,^^11^

Vaccination in children aged 5–11 years started in January 2022, after the Brazilian Health Regulatory Agency (ANVISA) approved Pfizer's mRNA vaccine (BNT162b2) for this age group. Despite the recommendation of specialized societies, vaccination started amid intense social controversy fueled by misinformation campaigns. The vaccination campaign also faced many operational difficulties and meager mass media campaigns of mobilization and communication about the importance of vaccinating children. These are among the many reasons which may account for the slow pace of vaccination in this age group, reaching national average coverage with first dose of only 22.7% by March 15th, 2022.^12^ Coverage levels in the different Brazilian states is very heterogeneous, varying from 3.7% up to 46.0% at the time.^12^

To evaluate the impact of vaccination against COVID-19 in children aged 5–11 years in the epidemiological dynamics of SARS-CoV-2 in Brazil, we conducted a modeling simulation study to estimate how many COVID-19 hospitalizations, deaths, and potential years of life lost could be averted, as well as the financial savings resulting from averted hospitalizations resulting from childhood vaccination. Moreover, we estimated the additional benefits of a child vaccination campaign that reaches a maximum pace compared to the currently observed pace of childhood vaccine administration in Brazil at the time of modeling.

## Methods

### Data used in the model

Anonymized information of individuals' vaccine status from the Information System of the National Immunization Program (SIPNI, https://opendatasus.saude.gov.br/dataset/covid-19-vacinacao) of Brazil and the Influenza Epidemiological Surveillance System (SIVEP-Gripe, https://opendatasus.saude.gov.br/dataset/srag-2020, https://opendatasus.saude.gov.br/dataset/srag-2021-e-2022) were used to calibrate the model. SIPNI yielded vaccine coverage rates by age group, dose type, and vaccine type by each one of the 27 states and federative unit of the country. The following population age sub-groups were considered: 1–4, 5–11, 12–17, 18–29, 30–39, 40–49, 50–59, 60–69, 70–79, 80–89, and 90+ years. We computed the coverage of vaccinated individuals according to dose (first, second, and booster doses) and vaccine product (BNT162b2, AZD1222, and CoronaVac), by state and age group. The population size for each age sub-group was estimated based on population projections made by the Brazilian Institute of Geography and Statistics.^5^ SIVEP-Gripe provided numbers of confirmed COVID-19 cases, hospitalizations and deaths in Brazil over time and by state. The time series data extracted from this database, extracted in January 26th, 2022, were corrected by nowcasting estimates in order to minimize the effects of delays in notification.^13^

To estimate the potential costs averted due to COVID-19 hospitalizations prevented by vaccination, we considered the average reimbursement cost of hospitalizations (considering both regular and intensive care hospitalization costs for children and adults) obtained from the Brazilian Hospital Information System (SIH-DATASUS)^14^ for November and December 2021. The average reimbursement values weighted by macro-region were obtained in Brazilian Reais (BRL) and then converted to International Dollars (Int$) considering the 2021 purchasing power (4102.37 Int$/hospitalization, 1 Int$ = 2.53 BRL).

### Model structure

We developed a SEIR-like model in discrete-time and stratified in age groups, considering the following scenarios of vaccine administration in 5–11 years age group: *i*) No vaccination; *ii*) Slow vaccination pace (current), considered as 250,000 vaccines doses administered per day nationally; *iii*) Maximum vaccination pace, considered as 1 million doses administered per day. The current vaccination pace was estimated considering information shared by the Ministry of Health and reported by the press on the number of vaccine doses distributed weekly by the National Immunization Program to the states and federative units. We assumed that the doses were evenly distributed over time. The maximum vaccination pace considered the true vaccine administration capacity of the National Immunization Program, based in previous child vaccination campaigns in the country.^15^, ^16^, ^17^ In this scenario, higher vaccine coverage rates could be reached earlier.

For the remaining population age groups (adolescent, adults, and elderly), we considered vaccine coverage per dose and vaccine product, by state obtained from SIPNI^12^ at 2022-01-02. In these groups, we assumed a fixed vaccine coverage rate during the 3-month time horizon, to explicitly estimate the incremental impact of child vaccination over the existing vaccination strategies targeting other age groups.

**Fig. 1:**
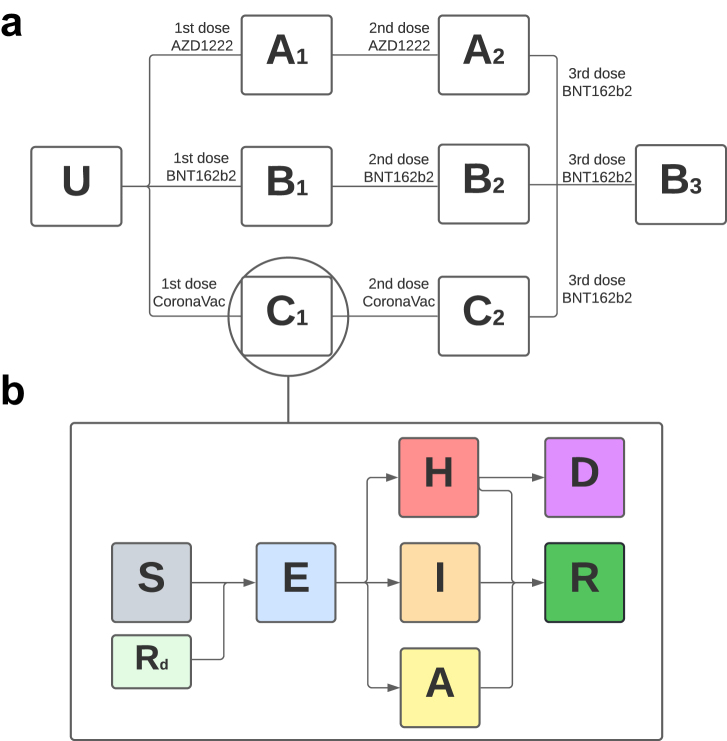
**Model structure.** Schematic compartmental structure of model, with each compartment replicated for each vaccine product and dose. For more information, please see the **Model** section in **Methods**. **(a)** Structure replication for each vaccine product and dose. **(b)** General compartmental structure of model.

The SEIR model used is constituted of the following compartments (Fig. 1b): Susceptibles (S), Exposed and infective (E), Asymptomatics (A), mild Symptomatics (I), Hospitalized (H), Recovered (R) and Deaths (D) due to COVID-19. We assume that Exposed individuals have reduced infectiousness in comparison to symptomatic individuals due to the incubation period, whereas Hospitalized individuals have reduced infectiousness due to isolation. We assume that Asymptomatic individuals have the same infectiousness as Symptomatic. We model the contacts of individuals between different age groups using the contact matrices estimated for the Brazilian population and our infection model in discrete-time (for a complete description please see Supplementary Methods). This structure simulates all the conditions of clinical and infectious progression possible relative to a SARS-CoV-2 infection. This structure is replicated considering vaccination, with each compartment using the corresponding parameters of vaccine effectiveness, by dose, against different endpoints for each one of the vaccine products in use in the country (BNT162b2, AZD1222, and CoronaVac), by age group as shown in Fig. 1a.

Our model considers a 90-day time horizon, between January 2022 and April 2022, period in which we model disease transmission and occurrence and hence estimated impact of children vaccination. During this period, omicron was the dominant circulating variant; individuals previously infected were assumed to be Recovered individuals at the beginning of the simulation having been infected mainly by Gamma and Delta variants.^18^ Equations are described in the Supplementary Methods.

### Model parameters

The effectiveness of the various vaccine products used in Brazil and the effectiveness of BNT162b2 vaccine in children aged 5–11 years, by dose, for each endpoint and age group, were obtained through a literature review (see Supplementary Figs. S1–S4). Vaccine effectiveness (VE) against infection were based on the studies by the New York Department of Health^19^ (for age group 5–11 years), Powell et al. (for 12 to 18 age group)^20^ and, for other ages, the studies by Andrews^4^ and Hung^21^ or Willet et al.^22^ VE estimates against COVID-19 hospitalization and death considered were those reported by Barnard et al.,^23^ from UKHSA,^24^ and Young Xu et al.^25^

We assumed no effectiveness of inactivated Sinovac© vaccine (CoronaVac) against omicron infection. For other outcomes (COVID-19 hospitalization and deaths), considering the limited available evidence, we assumed a reduction of 50% in effectiveness when compared to the estimated effectiveness of this vaccine against these outcomes for other variants.^26^

Point estimate and corresponding 95% confidence intervals (CI) of each vaccine effectiveness estimates were considered, when available. These values were used to parametrize a Beta distribution and then imputed to the model. We then sample each parameter a thousand times to provide a combination of effectiveness samples. In addition, we sample an assumed distribution of Recovered individuals with a mean of around 70% of the population (see Supplementary Methods). Even though we do not have prevalence estimates for each state of the country, seroprevalence estimates from blood donors in selected cities prior to vaccination and Gamma VOC epidemic wave shows that prevalence rates in the country were already high prior to the introduction of omicron VOC, supporting our assumptions.^18^

To account for the heterogeneity in disease dynamics, we fit the number of weekly new COVID-19 hospitalizations in each federative unit to an exponential function to estimate the hospitalization growth rate, afterwards, we sampled the growth rate assuming a normal distribution with deviance from the fitting procedure.

Finally, considering vaccine effectiveness parameters, initial prevalence at start of simulation and hospitalization growth rate, we compute the corresponding basic reproduction number (R_0_) of omicron infection using the Next Generation Matrix (NGM) method, adjusting the growth rate estimated from secondary surveillance data to the growth rate of the Jacobian of the model.^27^ Using the eigenvector associated with the largest eigenvalue of the Jacobian (i.e. the growth rate), we compute the proportion of individuals each compartment, also accounting for variation due to age groups, as described in the Supplementary Methods.

### Impact estimation and sensitivity analysis

Children vaccination impact was estimated in terms of COVID-19 hospitalizations and deaths averted, as well as resulting averted costs of hospital admissions, and potential years of life lost (YLL) averted. For this estimate, COVID-19 deaths which would occur without childhood vaccination were considered, and the average age of death due to COVID-19 in each age sub-group was subtracted from the average life expectancy at birth in Brazil (76 years^5^), and then multiplied to the total death count of each age group.

These outcomes were estimated for a period of 3 months after the start of vaccination (time horizon of the analysis). Thus, after estimating and projecting the expected number of hospitalizations and deaths from COVID-19, by age group, for each scenario, we estimated the avoided number of hospitalizations and deaths resulting from the vaccination of children between 5 and 11 years. We measured both the impact of vaccination for the population aged 5–11 years (direct effects of vaccination) and all other age groups (indirect effects of vaccination).

To account for variation and uncertainty on the main parameter values of the model, we conducted sensitivity analysis (Supplementary Table S5).

### Role of funding source

The funding source had no role in the design of this study during its execution, analyses, interpretation of the data, or decision to submit results.

## Results

**Fig. 2:**
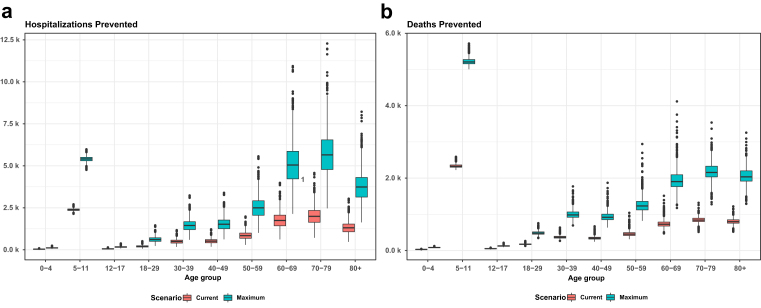
**Boxplot of the number of preventable events** by vaccination against COVID-19 in children, by age group. In **(a)** corresponding to preventable hospitalizations and **(b)** corresponding to preventable deaths by COVID-19.

In Fig. 2 and Table 1, we present the estimated averted events by age group, considering the two vaccination pace scenarios. In the three-month period of analysis, vaccinating children at the (current) slow pace of vaccination has the potential to prevent 1450 deaths [P2.5–P97.5 805–2360] and 9704 hospitalizations [P2.5–P97.5 6329–14,237] from COVID-19 for all age groups (Table 1). When we consider only the direct impact of the vaccine on children aged 5–11 years in the scenario of a slow vaccination pace, we found an impact of 180 avoided deaths [P2.5–P97.5 170–190] and 2389 avoided hospitalizations [P2.5–P97.5 2253–2553] (Table 1).

**Table 1:** Averted outcomes by vaccination scenario.

Outcome	Age group	Vaccination at slow pace (Thousands)	Vaccination at maximum pace (Thousands)
Hospitalization	All	9.7 [6.33–14.24]	26.56 [17.76–38.39]
Death	All	1.45 [0.8–2.36]	4.25 [2.38–6.89]
Hospitalization	5–11	2.39 [2.25–2.55]	5.4 [5.07–5.74]
Death	5–11	0.18 [0.17–0.19]	0.41 [0.38–0.44]

Considering vaccination at an maximum pace, with the administration of 1 million doses per day of vaccines against COVID-19 to children between 5 and 11 years old, the impact would be much higher, avoiding a total of 4251 COVID-19 deaths [P2.5–P97.5 2380–6894] and 26,560 hospitalizations [P2.5–P97.5 17,759–38,391] (Supplementary Table S1). If the speed of vaccination proceeded at maximum pace, it would be possible to avoid 412 deaths [P2.5–P97.5 385–437] and 5403 hospitalizations [P2.5–P97.5 5070–5744] due to COVID-19 in children aged between 5 and 11 years (Table 1).

Regarding the cost analysis, the hospitalizations avoided in all age groups would result in a cost reduction of Int$ 39,812,206 [P2.5-P97.5 22,208,828.80–25,521,822.60] considering vaccination at a slow pace, reaching Int$ 57,920,893.40 [P2.5–P97.5 54,922,182.90–62,430,000.90] for a maximum vaccination pace (Table 2). Considering only the reduction in costs related to averted hospitalizations in children aged 5–11 years, vaccination at a maximum pace could lead to a reduction in costs in the amount of Int$ 22,232,244.50 [P2.5–P97.5 21,751,540.90–23,313,930.30] (Table 2). In addition, we estimated a total of 24,750 [P2.5–P97.5 18,690–33,338] YLL prevented by a slow pace of vaccination (with 12,232 [P2.5–P97.5 11,548–12,952] YLL reduction corresponding to direct effect) and a larger effect of 66,568 [P2.5–P97.5 48,659–92,291] YLL could be prevented in an maximum pace (also observing a prevention of 27,982 [P2.5–P97.5 26,161–29,711] YLL on age group 5–11 years) (Table 3).

**Table 2:** Averted costs by vaccination scenario and age group.

Age group	Vaccination at slow pace (cost in Million Int$)	Vaccination at maximum pace (cost in Million Int$)
All	39.81 [25.97–58.41]	108.96 [72.86–157.5]
0–4	0.4 [0.22–0.65]	1.17 [0.7–1.8]
5–11	24.79 [23.38–26.5]	56.08 [52.62–59.62]
12–17	0.62 [0.34–1]	1.76 [1.08–2.7]
18–29	2.16 [1.12–3.56]	6.46 [3.54–10.43]
30–39	5.18 [2.74–8.42]	15.25 [8.63–24.06]
40–49	5.44 [2.85–8.85]	16.02 [9.1–25.24]
50–59	8.93 [4.65–14.63]	26.4 [14.72–41.95]
60–69	18.43 [9.75–30.14]	53.33 [30.9–83.77]
70–79	20.98 [11.22–34.09]	59.79 [35.3–93.06]
80+	13.79 [7.33–22.49]	39.41 [22.89–61.71]

**Table 3:** Potential years of life lost averted by vaccination scenario.

Age group	Vaccination at slow pace	Vaccination at maximum pace
All	24,750 [18,690–33,338]	66,568 [48,659–92,291]
5–11	12,232 [11,548–12952]	27,982 [26,162–29,712]

In sensitivity analyses we changed parameters of vaccine effectiveness (Supplementary Figs. S1–S3), usually considering lower precision and/or lower effectiveness estimates. Model results were robust and did not vary significantly (Supplementary Table S5). In the current/slow vaccination pace we have estimated that 1539 COVID-19 deaths [P2.5–P97.1308–1860] and 6160 [P2.5–P97.5352–7169] hospitalizations would be averted in all age groups, reaching higher impact with maximum vaccination pace of 4310 COVID-19 deaths [P2.5–P97.5 3639–5270] and 15,302 hospitalizations [P2.5-P97.5 13,091–18,442] averted. Estimated averted COVID-19 deaths and hospitalizations in children aged 5–11 years considering both slow and maximum vaccination paces did not change in sensitivity analysis.

## Discussion

Significant impact of COVID-19 vaccination in children was estimated in Brazil shortly after omicron VOC introduction, and during the initial increase and epidemic wave caused by this VOC in early 2022 in the country.

The comparison of the two vaccination scenarios reinforces that timing and efficiency of COVID-19 vaccination in children have significant impacts, with a maximum pace of vaccination resulting in a reduction of COVID-19 hospitalizations and deaths three times higher, when compared to slow pace of vaccination. The total number of hospitalizations and deaths preventable by vaccination in a period of three months in children between 5 and 11 years old has the same order of magnitude as the total number of COVID-19 deaths (308) and hospitalizations (6877) that occurred in this age group during the entire COVID-19 pandemic, from February 2020 to February 2022, in Brazil.

Assuming an estimated 20 million of children in Brazil, we estimated a total of 4250 prevented hospitalizations in three months, a higher estimative than the one reported by the US CDC study that estimated the benefits for every million doses of Pfizer-BioNTech COVID-19 vaccination in children 5–11 years of age using two incidence scenarios: scenario A “recent epidemiology data” from the week ending on September 9th, 2021 and scenario B “pandemic average data'' assuming an average for the entire pandemic through the week ending on October 16th, 2021. Prevented COVID-19 and hospitalization cases were estimated as, respectively, 58,204 and 226 in scenario A and 18,549 and 80 in scenario B.^8^ Such difference may be because the above CDC estimates were based on an epidemiological scenario previous to the introduction of VOC omicron. More recently, Borchering et al.,^28^ using a robust methodology based on these CDC models, modeled the potential benefit of vaccinating children 5–11 years in a scenario of omicron dominance estimating approximately 47,000 COVID-19 hospitalizations averted. As this modeling was conducted before omicron predominance, the authors assumed higher infection transmission rates and same vaccine effectiveness (when compared to delta VOC), and incorporated the effects of non-pharmacological interventions (NPI) in place at the time in the US. In contrast, at the time of our modeling, Brazil had already relaxed most NPI previously in place in the country, as well mandatory mask mandates in public places,^29^ so these were not considered in our modeling. Furthermore, we did update and considered more recent evidence of lower VE effectiveness against omicron VOC (for selected vaccines and against specific outcomes) available at the time. This might explain the different magnitude in the estimated impact of children vaccination reported.

The United Kingdom Joint Commission on Vaccination and Immunization (JCVI) estimated that children vaccination might prevent 98 hospitalizations for every million administered doses in 20 weeks, and thus 1760 hospitalizations in the period. In contrast, JCVI considered higher protection post-infection and lower vaccine effectiveness as parameters to modeling. Contrast among these results and ours may be justified to various factors, including differences in local disease epidemiology, vaccine estimates of effectiveness, and modeling methods. In addition, they also report a small indirect effect on hospitalizations and deaths as a result of childhood vaccination, a finding very similar to our results.

In relation to indirect effects (i.e prevention of deaths and hospital admissions on adults), we hypothesized that it could be related to the proportion of people vaccinated with booster doses, (since this effect is greater in federative units with lower proportions of booster dose as seen on Supplementary Figs. S5 and S6) and found a moderate/weak, non-significant correlation between indirect impact and 3rd dose coverage in the 27 states in Brazil (Supplementary Fig. S7), noteworthy, this correlation may be explained to several reasons, including socioeconomic disparities and vaccine distribution, as observed by Liu et al. when analyzing in hospital mortality in Brazilian federative units.^30^ Even so, an indirect effect on hospitalizations of a child vaccination strategy for influenza was found in previous mathematical modeling studies.^31^

The economic impact was also significant (about 40 million Int$ for slow vaccination pace and up to 109 Million Int$ in maximum pace), albeit underestimated as it considered only averted hospitalization costs. Furthermore, since the reimbursement value paid by the National Health System to hospitals, obtained from SIH-DATASUS was considered to estimate averted costs, the cost of each hospitalization is also underestimated, as reimbursed costs have been demonstrated to be much lower than real costs of hospitalizations using micro-costing methodology, especially in patients with SARI due to COVID-19.^32^ For example, Miethke-Morais et al. estimated the average cost of one COVID-19 hospitalization at Int$ 12,000 (equivalent to US$), a value much higher than the reimbursed value of Int$ 4102.37 we considered in our estimates.

Our study has several limitations. First, in order to assess the effects of vaccinating children, we assumed that the vaccination coverage in adults remained constant throughout our simulations. Although this assumption may lead to an increased number of expected hospitalizations and deaths for these age groups, incorporating increasing vaccine coverage in adults would not qualitatively alter our results, and imply only minor quantitative changes in our estimates. Since the end of 2021, the pace of vaccination in adults has significantly decreased, especially in older age groups, the population at greater risk of hospitalization and death.^12^ From January 2022, the majority of the population from this age group willing to adhere to the vaccination campaign have already been vaccinated with 3 doses. For older adults (more than 60 years), booster shots were available since October 2021.^12^ Furthermore, adults below 60 years who received the second or booster doses between January and March 2022 would have a lower risk of developing severe disease.^4^^,^^24^ Thus, a small increase in the vaccination coverage for these groups for the analyzed period would likely have little impact in our estimates and main conclusions.

Second, the impact estimated by our model considers only a fixed period of time. The decision to assess the impact for a short-term period relies on the understanding that the longer the projection time of the epidemiological dynamics, the greater the uncertainty.^33^ In addition, high disease burden in children would likely occur during a period of intense circulation and epidemic wave of the newly introduction and predominance of the omicron VOC, which is not expected to last more than 3 months, as it was later demonstrated. However, limiting the estimation for a 3-month period does not capture the benefit of vaccinating children in the context of continuing transmission of COVID-19 as the most plausible scenario includes the persistence of transmission in different locations and populations, albeit at endemic level.^34^ Although there is great uncertainty regarding transmission rates in the medium and long-term, even the maintenance of endemic levels of transmission can present a continuous risk to the health of children, in addition to the risk of new epidemic waves and increased levels of transmission due to the eventual introduction of new variants^34^ or untimely relaxation of adherence to non-pharmacological interventions (NPIs).^35^

Thus, we conclude that vaccination in the 5–11 age group has significant potential to reduce the impact of the omicron variant in terms of hospitalizations, deaths and associated costs. The impact of vaccination can be significantly greater if the vaccination pace is higher, achieving faster vaccination of this population and higher vaccine coverage in a shorter period. Childhood vaccination can result in significant impact and should be introduced, particularly in settings with intense viral circulation in this age group, and after having achieved high vaccine coverage rates in priority groups for vaccination.

## Contributors

Muller GC and Ferreira LS contributed equally as first authors, summarizing data as well as elaborating the first draft of the manuscript. Muller GC and Almeida GB searched and summarized the parametrization for vaccine effectiveness. Ferreira LS, Mesias Campos FE, Borges, ME, Poloni S, Coutinho RM and Kraenkel, RA developed the model and analyzed data. Camey SA, Kuchenbecker RS and Toscano CM developed theoretical structure, revised VE parameters, discussed results and, and revised the manuscript. Muller GC, Ferreira LS, Almeida GB, Mesias Campos FE, Borges ME, Coutinho RM, Kraenkel RA, Simon LM, Bagattini AM, Rosa MQM, Diniz Filho JAF, Camey SA, Kuchenbecker RS and Toscano CM contributed for manuscript elaboration, theoretical discussion and revision.

## Data sharing statement

The datasets, original or processed, and code used in this work are available at: https://github.com/covid19br/child_vac_omicron. All scripts were custom built in R language.

## Editor note

*The Lancet* Group takes a neutral position with respect to territorial claims in published maps and institutional affiliations.

## Declaration of interests

MEB received payment fees for consulting service for work on database management in the municipality of Florianópolis, funded by 10.13039/100011893PAHO/10.13039/100004423WHO (contract number CON21-00014067). All authors declare no conflict of interest.
