# Supplementary material for: Modeling the impact of child vaccination (5–11 y) on overall COVID-19 related hospitalizations and mortality in a context of omicron variant predominance and different vaccination coverage paces in Brazil
Source: Lancet Reg Health Am. 2022 Nov 17;17:100396. doi: 10.1016/j.lana.2022.100396 (PMC9678377; doi:10.1016/j.lana.2022.100396)
Supplement: Supplementary Material S1 [file mmc1.pdf]

Modeling the impact of child vaccination (5 to 11 y) on overall COVID-19 related hospitalizations and mortality in a context of omicron variant predominance and different vaccination coverage paces in Brazil

Supplementary Material

Gabriel Cardozo Müller[1,*], Leonardo Souto Ferreira[2,3,*], Felipe Ernesto Mesias Campos[2,4], Marcelo Eduardo Borges[2], Gabriel Berg de Almeida[5], Silas Poloni[2,3], Lorena Mendes Simon[6], Angela Maria Bagattini[7], Michelle Quarti Machado da Rosa[7], José Alexandre Felizola Diniz Filho[1,4], Roberto André Kraenkel[1,2], Renato Mendes Coutinho[2,8], Suzy Alves Camey[7], Ricardo de Souza Kuchenbecker[6], and Cristiana Maria Toscano[4]

[1]*Programa de Pós-graduação em Epidemiologia, Faculdade de Medicina - Universidade Federal do Rio Grande do Sul, Campus Saúde, Rua Ramiro Barcelos, 2400, 2º andar, Floresta, Porto Alegre - RS, Brazil*
[2]*Observatório Covid-19 BR*
[3]*Instituto de Física Teórica - Universidade Estadual Paulista, Rua Dr. Bento Teobaldo Ferraz, 271, Várzea da Barra Funda, São Paulo - SP, Brazil*
[4]*Programa de Pós-Graduação em Ecologia, Instituto de Biociências – Universidade de São Paulo, Rua do Matão, 321 - Trav. 14 São Paulo – SP, Brazil*
[5]*Departamento de Infectologia, Faculdade de Medicina de Botucatu - Universidade Estadual Paulista, Av. Prof. Mário Rubens Guimarães Montenegro, s/n, Botucatu, São Paulo, Brazil.*
[6]*Departamento de Ecologia, Instituto de Ciências Biológicas, Universidade Federal de Goiás, CP 131, Goiânia, Goiás, Brazil*
[7]*Departamento de Saúde Coletiva, Instituto de Patologia Tropical e Saúde Pública, Universidade Federal de Goiás, Rua 235, s/n.º, Setor Leste Universitário, Goiânia, Goiás; Brazil*
[8]*Centro de Matemática, Computação e Cognição - Universidade Federal do ABC, Avenida dos Estados, 5001, Bangú, Santo André - SP, Brazil*
[9]*Instituto de Matemática e Estatística, Departamento de Estatística - Universidade Federal do Rio Grande do Sul, Avenida Bento Gonçalves, 9500, Agronomia, Porto Alegre - RS, Brazil*
[10]*Hospital de Clínicas de Porto Alegre, Rua Ramiro Barcelos, 2350, Santa Cecília, Porto Alegre - RS, Brazil*
[*]*Gabriel Cardozo Müller and Leonardo Souto Ferreira contributed equally as first authors*

October 8, 2022

1 Introduction

In this material, we describe the methodology used in the paper. The code is available at https://github.com/covid19br/child_vac_omicron. In section 2.1 we describe how we account two strains in the model. In section 2.2 we describe the basic epidemiological model. In section 3 we describe how we calculate the Effective Reproduction Number, growth rate and initial conditions from hospitalization data. In section 4 we describe the parameters used in the model, together with sources. Finally, in section 5 we show additional results concerning total number of events, sensitivity analysis, and a possible explanation for the heterogeneity between states.

2 Model

2.1 Modelling competition between variants

For simplicity, suppose that we have a simple SIR model with two strains named D and O. Then the probability of a susceptible individual not being infected between t and $t+1$ is given by:

$$P(S|I_D, I_O) = \exp\left(-\beta_D I_D - \beta_O I_O\right) \tag{1}$$

where β is the probability of infection given a contact.

Conversely, the probability of becoming infected by ANY of the strains is given by:

$$P(I|I_D, I_O) = 1 - \exp\left(-\beta_D I_D - \beta_O I_O\right) \tag{2}$$

Since being infected by one strain precludes infection by the other strain, the probability of being infected by an specific strain is given by the rate of events of infection of each strain, as given by the Gillespie algorithm. Thus, we have:

$$P(I_D|I_D, I_O) = \frac{\beta_D I_D}{\beta_D I_D + \beta_O I_O}\left(1 - \exp\left(-\beta_D I_D - \beta_O I_O\right)\right) \tag{3}$$

$$P(I_O|I_D, I_O) = \frac{\beta_O I_O}{\beta_D I_D + \beta_O I_O}(1 - \exp(-\beta_D I_D - \beta_O I_O)) \tag{4}$$

Therefore, a complete two-strain SIR discrete time model would be given by:

$$S^{t+1} = \exp(-\beta_D I_D - \beta_O I_O) S^t \tag{5}$$

$$I_D^{t+1} = \frac{\beta_D I_D}{\beta_D I_D + \beta_O I_O}(1 - \exp(-\beta_D I_D - \beta_O I_O)) S^t + (1-\nu) I_D^t \tag{6}$$

$$I_O^{t+1} = \frac{\beta_O I_O}{\beta_D I_D + \beta_O I_O}(1 - \exp(-\beta_D I_D - \beta_O I_O)) S^t + (1-\nu) I_O^t \tag{7}$$

$$R_D^{t+1} = \nu I_D^t + R_D^T \tag{8}$$

$$R_O^{t+1} = \nu I_O^t + R_O^T \tag{9}$$

2.2 Epidemiological model

Following Diekmann et al. [11], we construct a discrete-time SEIR-like model. We assume that a susceptible individual (S) has a probability p of being infected. If a infection occurs, the individual transits to the exposed (E), pre-symptomatic, compartment. After the incubation period, the individual can transit to hospitalized (H), mildly symptomatic (I) or asymptomatic (A) compartments. If the individual is hospitalized, the possible outcomes are recovery (R) or death (D), with the respective compartments. In the case of asymptomatic and mildly symptomatic we assume recovery as the only possibility.

As some parameters can be described as rates $(1/t)$, we assume (as in typical SEIR-like models) an exponential distribution of time to transition. For example, the incubation period γ, can be rewritten as the probability of a pre-symptomatic individual exiting the Exposed compartment, given by $1 - \exp(-1/\gamma)$, as follows from the Gillespie algorithm.

Our model is age-structured. Since we aim to evaluate vaccination in children, we use specific age bins, those being $S = (S_{0-4}, S_{5-11}, S_{12-17}, S_{18-29}, S_{30-39}, S_{40-49}, \ldots, S_{80+})^T$, and for other classes in this way as well, but we drop those indexes for readability. Besides the age indexes, this model possess three more indexes, $j = \{u =$ unvaccinated$, v =$ vaccinated with one dose$, w =$ vaccinated with two doses$, b =$ vaccinated with booster dose$\}$, $k = \{A =$ AZD1222$, P =$ BNT16b2 (Pfizer's vaccine)$, C =$ CoronaVac$\}$ and $x = \{d =$ Delta$, o =$ Omicron$\}$. Since we assume that vaccinated individuals have equal transmissibility compared to unvaccinated individuals, consider $E := \sum_{j,k} E_{j,k,x}$ (not summed over ages), as well as the other classes. Thus, number of (alive) individuals is given by $N = S + E + I + A + H + R$. We use contact matrices between ages from Prem et al. [24]. The total number of contacts with infectious individuals per age and variant is then given by:

$$C_d = \hat{c}(I_d + \omega E_d + \omega_a A_d + \omega_s H_d)/N \tag{10}$$

$$C_o = \hat{c}(I_o + \omega E_o + \omega_a A_o + \omega_s H_o)/N \tag{11}$$

Finally, our model is given by:

Unvaccinated

$$S_u^{t+1} = \exp(-\beta_d C_d - \beta_o C_o)\left(1 - \sum_k V_{svk}^t\right) S_u^t \tag{12a}$$

$$\begin{aligned} E_{ux}^{t+1} = {} & \frac{\beta_x C_x}{\beta_d C_d + \beta_o C_o}(1 - \exp(-\beta_d C_d - \beta_o C_o))\left(1 - \sum_k V_{svkx}^t\right) S_u^t \\ & + \frac{\rho_{dx}\beta_x C_x}{\rho_{dd}\beta_d C_d + \rho_{do}\beta_o C_o}(1 - \exp(-\rho_{dd}\beta_d C_d - \rho_{do}\beta_o C_o))\left(1 - \sum_k V_{rvkx}^t\right) R_{ud}^t \\ & + \frac{\rho_{ox}\beta_x C_x}{\rho_{od}\beta_d C_d + \rho_{oo}\beta_o C_o}(1 - \exp(-\rho_{od}\beta_d C_d - \rho_{oo}\beta_o C_o))\left(1 - \sum_k V_{rvkx}^t\right) R_{uo}^t \\ & + (1 - \gamma_{ux}) E_{ux}^t \end{aligned} \tag{12b}$$

$$I_{ux}^{t+1} = (1 - \sigma_{ux})(1 - \alpha_{ux})\gamma_x E_{ux}^t + (1 - \nu) I_{ux}^t \tag{12c}$$

$$A_{ux}^{t+1} = (1 - \sigma_{ux})\alpha_{ux}\gamma_x E_{ux}^t + (1 - \nu) A_{ux}^t \tag{12d}$$

$$H_{ux}^{t+1} = \sigma_{ux}\gamma_x E_{ux}^t + (1 - \nu_s) H_{ux}^t \tag{12e}$$

$$
\begin{aligned}
R_{ux}^{t+1} &= \exp(-\rho_{dd}\beta_d C_d - \rho_{do}\beta_o C_o)\left(1 - \sum_k V_{rvkx}^t\right) R_{ux}^t \\
&\quad + (1-\mu)\nu_s H_{ux}^t + \nu A_{ux}^t + \nu I_{ux}^t && \text{(12f)} \\
D_{ux}^{t+1} &= D_{ux}^t + \mu_{ux}\nu_s H_{ux}^t && \text{(12g)}
\end{aligned}
$$

1 Dose

$$
\begin{aligned}
S_{vk}^{t+1} &= \exp(-\beta_d C_d - \beta_o C_o) V_{svk}^t S_u^t \\
&\quad + \exp(-\beta_{vkd} C_d - \beta_{vko} C_o)\left(1 - V_{swk}^t\right) S_{vk}^t && \text{(12h)} \\
E_{vkx}^{t+1} &= \frac{\beta_x C_x}{\beta_d C_d + \beta_o C_o}(1 - \exp(-\beta_d C_d - \beta_o C_o)) V_{svk}^t S_u^t \\
&\quad + \frac{\rho_{dx}\beta_x C_x}{\rho_{dd}\beta_d C_d + \rho_{do}\beta_o C_o}(1 - \exp(-\rho_{dd}\beta_d C_d - \rho_{do}\beta_o C_o)) V_{rvkx}^t R_{ud}^t \\
&\quad + \frac{\rho_{ox}\beta_x C_x}{\rho_{od}\beta_d C_d + \rho_{oo}\beta_o C_o}(1 - \exp(-\rho_{od}\beta_d C_d - \rho_{oo}\beta_o C_o)) V_{rvkx}^t R_{uo}^t \\
&\quad + \frac{\beta_{vkx}\beta_x C_x}{\beta_{vkd} C_d + \beta_{vko} C_o}(1 - \exp(-\beta_{vkd} C_d - \beta_{vko} C_o))\left(1 - V_{swkx}^t\right) S_{vk}^t && \text{(12i)} \\
&\quad + \frac{\rho_{dx}\beta_{vkx} C_x}{\rho_{dd}\beta_{vkd} C_d + \rho_{do}\beta_{vko} C_o}(1 - \exp(-\rho_{dd}\beta_{vkd} C_d - \rho_{do}\beta_{vko} C_o))\left(1 - V_{rwkd}^t\right) R_{vkd}^t \\
&\quad + \frac{\rho_{ox}\beta_{vkx} C_x}{\rho_{od}\beta_{vkd} C_d + \rho_{oo}\beta_{vko} C_o}(1 - \exp(-\rho_{od}\beta_{vkd} C_d - \rho_{oo}\beta_{vko} C_o))\left(1 - V_{rwko}^t\right) R_{vko}^t \\
&\quad + (1-\gamma_x) E_{vkx}^t \\
I_{vkx}^{t+1} &= (1-\sigma_{vkx})(1-\alpha_{vkx})\gamma_x E_{vkx}^t + (1-\nu) I_{vkx}^t && \text{(12j)} \\
A_{vkx}^{t+1} &= (1-\sigma_{vkx})\alpha_{vkx}\gamma_x E_{vkx}^t + (1-\nu) A_{vkx}^t && \text{(12k)} \\
H_{vkx}^{t+1} &= \sigma_{vkx}\gamma_x E_{vkx}^t + (1-\nu_s) H_{vkx}^t && \text{(12l)} \\
R_{vkx}^{t+1} &= (1-\mu_{vkx})\nu_s H_{vkx}^t + \nu A_{vkx}^t + \nu I_{vkx}^t \\
&\quad + \exp(-\rho_{dd}\beta_{vkd} C_d - \rho_{do}\beta_{vko} C_o)(1 - V_{rwkx}^t) R_{vkx} && \text{(12m)} \\
&\quad + \exp(-\rho_{dd}\beta_d C_d - \rho_{do}\beta_o C_o) V_{rvkx}^t R_{ux} \\
D_{vkx}^{t+1} &= D_{vkx}^t + \mu_{vkx}\nu_s H_{vkx}^t && \text{(12n)}
\end{aligned}
$$

2 Doses

$$
\begin{aligned}
S_{wk}^{t+1} &= \exp(-\beta_{vkd} C_d - \beta_{vko} C_o) V_{wk}^t S_{vk}^t \\
&\quad + \exp(-\beta_{wkd} C_d - \beta_{wko} C_o)(1 - V_{bp}^t) S_{wk}^t && \text{(12o)} \\
E_{wkx}^{t+1} &= \frac{\beta_{vkx} C_x}{\beta_{vkd} C_d + \beta_{vko} C_o}(1 - \exp(-\beta_{vkd} C_d - \beta_{vko} C_o)) V_{swk}^t S_{vk}^t \\
&\quad + \frac{\rho_{dx}\beta_{vkx} C_x}{\rho_{dd}\beta_{vkd} C_d + \rho_{do}\beta_{vko} C_o}(1 - \exp(-\rho_{dd}\beta_{vkd} C_d - \rho_{do}\beta_{vko} C_o)) V_{rwkd}^t R_{vkd}^t \\
&\quad + \frac{\rho_{ox}\beta_{vkx} C_x}{\rho_{od}\beta_{vkd} C_d + \rho_{oo}\beta_{vko} C_o}(1 - \exp(-\rho_{od}\beta_{vkd} C_d - \rho_{oo}\beta_{vko} C_o)) V_{rwko}^t R_{vko}^t \\
&\quad + \frac{\beta_{wkx}\beta_x C_x}{\beta_{wkd} C_d + \beta_{wko} C_o}(1 - \exp(-\beta_{wkd} C_d - \beta_{wko} C_o))\left(1 - V_{sbp}^t\right) S_{wk}^t && \text{(12p)} \\
&\quad + \frac{\rho_{dx}\beta_{wkx} C_x}{\rho_{dd}\beta_{wkd} C_d + \rho_{do}\beta_{wko} C_o}(1 - \exp(-\rho_{dd}\beta_{wkd} C_d - \rho_{do}\beta_{wko} C_o))\left(1 - V_{rbpd}^t\right) R_{wkd}^t \\
&\quad + \frac{\rho_{ox}\beta_{wkx} C_x}{\rho_{od}\beta_{wkd} C_d + \rho_{oo}\beta_{wko} C_o}(1 - \exp(-\rho_{od}\beta_{wkd} C_d - \rho_{oo}\beta_{wko} C_o))\left(1 - V_{rbpd}^t\right) R_{wko}^t \\
&\quad + (1-\gamma_d) E_{wkx}^t \\
I_{wkx}^{t+1} &= (1-\sigma_{wkx})(1-\alpha_{wkx})\gamma_x E_{wkx}^t + (1-\nu) I_{wkx}^t && \text{(12q)} \\
A_{wkx}^{t+1} &= (1-\sigma_{wkx})\alpha_{wkx}\gamma_x E_{wkx}^t + (1-\nu) A_{wkx}^t && \text{(12r)} \\
H_{wkx}^{t+1} &= \sigma_{wkx}\gamma_x E_{wkx}^t + (1-\nu_s) H_{wkx}^t && \text{(12s)} \\
R_{wkx}^{t+1} &= (1-\mu_{wkx})\nu_s H_{wkx}^t + \nu A_{wkx}^t + \nu I_{wkx}^t
\end{aligned}
$$

$$
\begin{aligned}
&+\exp(-\rho_{dd}\beta_{wkd}C_d-\rho_{do}\beta_{wko}C_o)(1-V^t_{rbpx})R_{wkd} &\quad (12t)\\
&+\exp(-\rho_{dd}\beta_{vkd}C_d-\rho_{do}\beta_{vko}C_o)V^t_{rwkx}R_{vkd}\\
D^{t+1}_{wkx} &= D^t_{wkx}+\mu_{wkx}\nu_s H^t_{wkx} &\quad (12u)
\end{aligned}
$$

Booster dose

$$
\begin{aligned}
S^{t+1}_{bp} &= \sum_k \exp(-\beta_{wkd}C_d-\beta_{wko}C_o)V^t_{sbp}S^t_{wk}\\
&+\exp(-\beta_{bpd}C_d-\beta_{bpo}C_o)S^t_{bp} &\quad (12v)\\
E^{t+1}_{bpx} &= \sum_k \frac{\beta_{wkxC_x}}{\beta_{wkd}C_d+\beta_{wko}C_o}(1-\exp(-\beta_{wkd}C_d-\beta_{wko}C_o))V^t_{sbp}S^t_{wk}\\
&+\sum_k \frac{\rho_{dx}\beta_{wkx}C_x}{\rho_{dd}\beta_{wkd}C_d+\rho_{do}\beta_{wko}C_o}(1-exp(-\rho_{dd}\beta_{wkd}C_d-\rho_{do}\beta_{wko}C_o))V^t_{rbpd}R^t_{wkd}\\
&+\sum_k \frac{\rho_{ox}\beta_{wkx}C_x}{\rho_{od}\beta_{wkd}C_d+\rho_{oo}\beta_{wko}C_o}(1-exp(-\rho_{od}\beta_{wkd}C_d-\rho_{oo}\beta_{wko}C_o))V^t_{rbpo}R^t_{wko}\\
&+\frac{\beta_{bpx}C_x}{\beta_{bpd}C_d+\beta_{bpo}C_o}(1-\exp(-\beta_{bpd}C_d-\beta_{bpo}C_o))S_{bp} &\quad (12w)\\
&+\frac{\rho_{dx}\beta_{bpx}C_x}{\rho_{dd}\beta_{bpd}C_d+\rho_{do}\beta_{bpo}C_o}(1-\exp(-\rho_{dd}\beta_{bpd}C_d-\rho_{do}\beta_{bpo}C_o))R_{bpd}\\
&+\frac{\rho_{ox}\beta_{bpx}C_x}{\rho_{od}\beta_{bpd}C_d+\rho_{oo}\beta_{bpo}C_o}(1-\exp(-\rho_{od}\beta_{bpd}C_d-\rho_{oo}\beta_{bpo}C_o))R_{bpo}\\
&+(1-\gamma_x)E_{bpx}\\
I^{t+1}_{bpx} &= (1-\sigma_{bpx})(1-\alpha_{bpx})\gamma_x E^t_{bpx}+(1-\nu)I^t_{bpx} &\quad (12x)\\
A^{t+1}_{bpx} &= (1-\sigma_{bpx})\alpha_{bpx}\gamma_x E^t_{bpx}+(1-\nu)A^t_{bpx} &\quad (12y)\\
H^{t+1}_{bpx} &= \sigma_{bpx}\gamma_x E^t_{bpx}+(1-\nu_s)H^t_{bpx} &\quad (12z)\\
R^{t+1}_{bpx} &= (1-\mu_{bpx})\nu_s H^t_{bpx}+\nu A^t_{bpx}+\nu I^t_{bpx}\\
&+\sum_k \exp(-\rho_{dd}\beta_{wkd}C_d-\rho_{do}\beta_{wko}C_o)V^t_{rbpd}R_{wkd} &\quad (12aa)\\
&+\exp(-\rho_{dd}\beta_{wkd}C_d-\rho_{do}\beta_{wko}C_o)R_{bpd}\\
D^{t+1}_{bpx} &= D^t_{bpx}+\mu_{bpx}\nu_s H^t_{bpx} &\quad (12ab)
\end{aligned}
$$

where k accounts for vaccine type and V^t_{jkx} is the proportion of individuals that have (or have not) been infected by variant x that receive the j dose of the said vaccine (Notice that V is proportional to S and R, but it is not written explicitly). We provide a description of each compartment and index used in the model in Table S1. The quantity of doses that are allocated for first or second dose are done as in [14].

We only consider vaccination of 5 to 11 years old in the model, and they do not receive booster doses (only 1 and 2 doses), as pediatric boosters were not approved in Brazil at the time of this study. While the model is flexible enough to deal with vaccinating all ages appropriately, we made the choice of not vaccinating adult individuals because it is difficult to predict what would be the vaccine uptake in this age group.

Usually, the vaccine allocation would also depend on the age of the individuals, but since we only vaccinate children from 5 to 11 years old, this is not necessary (but the model is flexible enough to deal with this). And the parameters after vaccination are written as done by Ferreira et al. [14]:

$$
\begin{aligned}
\beta_{jkx} &= (1-\varepsilon_{\beta,jkx})\beta_x\\
\alpha_{jkx} &= 1-(1-\varepsilon_{\alpha,jkx})(1-\alpha_x) &\quad (13)\\
\sigma_{jkx} &= (1-\varepsilon_{\sigma,jkx})\sigma_x\\
\mu_{jkx} &= (1-\varepsilon_{\mu,jkx})\mu_x
\end{aligned}
$$

where j denotes the vaccination status (v = 1 dose, w = 2 doses, b = booster dose), k denotes the vaccine type (A = AZD1222, B = BNT162b2, C = CoronaVac), and x the variant causing infection (d = Delta, o = Omicron) (See also Table S1). The values of each parameter (in absence of vaccine) are given in Tables S2 and S3.

We use the method developed in Ferreira et al. [14] (Supplementary Material) to remove multiplicative effects of effectiveness of vaccines. Since this depends non trivialy on the combination of effectiveness values, and these are sampled during simulation, we do not provide a table to the protection parameters after the mathematical treatment. But

the algorithm that calculates these values is available in the code repository provided with this material.

Compartment	Description
S	Susceptible individuals (i.e., no infection)
E	Exposed individuals, that may or may not present symptoms as the infection progress
I	Mild symptom infected individuals, that recover after some time
A	Asymptomatic infected individuals, that recover after some time
H	Hospitalized infected individuals, that may recover or die after some time
R	Recovered individuals, that may be reinfected in the future
D	Deceased individuals
**Index**	**Description**
u	Unvaccinated individuals
j	Index denoting vaccination state (v, w, b), as described below
v	Vaccinated individuals with only 1 dose
w	Vaccinated individuals with 2 doses
b	Vaccinated individuals with 2 doses + booster
k	Vaccine type (A = AZD1222, B = BNT162b2,C = CoronaVac)
x	Variant that caused infection (d = Delta, o = Omicron)

Table S1: Description of each compartment and index considered in the model.

3 Next Generation Matrix and Initial Conditions

In order to calculate the basic and effective reproduction numbers of the model we follow Allen and Van den Driessche [3] work. For that, we define $\mathbf{x}^t$ as the vector containing all infected classes, that is, all of $E_{a,j,k,x}$, $I_{a,j,k,x}$, $A_{a,j,k,x}$ and $H_{a,j,k,x}$, at time t and $\mathbf{y}^t$ as the vector containing the susceptible, recovered and deceased at time t. With it, we write our model as

$$\begin{cases} \mathbf{x}^{t+1} &= \mathbf{G}(\mathbf{x}^t, \mathbf{y}^t) \\ \mathbf{y}^{t+1} &= \mathbf{M}(\mathbf{x}^t, \mathbf{y}^t). \end{cases} \tag{14}$$

Suppose now that $\mathbf{G}$ can be written as a sum of two main processes, one given by $\mathbf{F}$, the new infections that survive from t to $t+1$ and the other one given by $\mathbf{T}$, the transitions between different infected classes, and also recoveries and deaths that happen from t to $t+1$. This is a classic decomposition in matrix population models, see, for instance, chapter five in Caswell [8]. Let $\hat{F}(\bar{\mathbf{x}}, \bar{\mathbf{y}})$ be the linearized matrix of $\mathbf{F}$ around a given equilibrium $(\bar{\mathbf{x}}, \bar{\mathbf{y}})$, and the same for $\hat{T}(\bar{\mathbf{x}}, \bar{\mathbf{y}})$ and $\hat{M}(\bar{\mathbf{x}}, \bar{\mathbf{y}})$. the system linearized around $(\bar{\mathbf{x}}, \bar{\mathbf{y}})$ is

$$\begin{cases} \mathbf{x}^{t+1} &= \mathbf{G}(\bar{\mathbf{x}}, \bar{\mathbf{y}}) + (\hat{F}(\bar{\mathbf{x}}, \bar{\mathbf{y}}) + \hat{T}(\bar{\mathbf{x}}, \bar{\mathbf{y}}))\mathbf{x}^t \\ \mathbf{y}^{t+1} &= \mathbf{H}(\bar{\mathbf{x}}, \bar{\mathbf{y}}) + \hat{H}(\bar{\mathbf{x}}, \bar{\mathbf{y}})\mathbf{y}^t \end{cases} \tag{15}$$

Note that around the given equilibrium, the number of new infections is given by the the $(\hat{F}(\bar{\mathbf{x}}, \bar{\mathbf{y}}) + \hat{T}(\bar{\mathbf{x}}, \bar{\mathbf{y}}))$ terms. If the equilibrium we linearize around is the disease free equilibrium (DFE), that is $(\bar{\mathbf{x}}, \bar{\mathbf{y}}) = (\mathbf{0}, \mathbf{y}_0)$, $\mathbf{y}_0$ the non-infected populations at DFE, we have $\mathbf{G}(0, \mathbf{y}_0) = 0$. With it, we define the basic reproduction number, R_0, as

$$R_0 = \rho \left\{ \hat{F}(\mathbf{0}, \mathbf{y}_0)(\mathbb{1} + \hat{T}(\mathbf{0}, \mathbf{y}_0))^{-1} \right\}, \tag{16}$$

where $\rho(\cdot)$ is the notation for the dominant eigenvalue, and $\hat{F}(\mathbf{0}, \mathbf{y}_0)(\mathbb{1} + \hat{T}(\mathbf{0}, \mathbf{y}_0))^{-1}$ is the next generation matrix (NGM).

Note now that the problem for $\mathbf{x}^t$ at DFE is

$$\mathbf{x}^{t+1} = (\hat{F}(\mathbf{0}, \mathbf{y_0}) + \hat{T}(\mathbf{0}, \mathbf{y_0}))\mathbf{x}^t, \tag{17}$$

and we write a solution as a linear combination of the eigenvectors, $\mathbf{q}_i$, and correspondent eigenvalues, r_i, of $(\hat{F}(\mathbf{0}, \mathbf{y_0}) + \hat{T}(\mathbf{0}, \mathbf{y_0}))$ as

$$\mathbf{x}^t = \sum_i \phi_i r_i^t \mathbf{q}_i. \tag{18}$$

The r_i can be ordered, that is $r^* = r_1 > r_2 > ... > r_D$, D the total number of infected classes. This way, for large t, $(r_i/r^*)^t \approx 0$ for $i > 2$ and we can write

$$\mathbf{x}^t = r^{*t} \sum_i \phi_i \left(\frac{r_i}{r^*} \right)^t \mathbf{q}_i \approx \phi^* r^{*t} \mathbf{q}^*. \tag{19}$$

To estimate proper values for the β_i coefficients we use a minimizing algorithm for r^*, the dominant eigenvalue in equation (19). Say that the observed growth coefficient is r_{obs} and for a given set of β_i, let's call it $B_j = (\beta_d^{(j)}, \beta_o^{(j)}, ...)$,

we find $r^*(B_j)$, the dominant eigenvalue of $\hat{F}+\hat{T}$ calculated with set B_j, and also $R_0(B_j)$, the basic reproduction number calculated with this same set. We then minimize the quantity $\|r(B_j)-r_{obs}\|$ by iterating the set B_j such that

$$B_j = \frac{R_0(B_{j-2})}{R_0(B_{j-1})} B_{j-1}, \tag{20}$$

where B_1 and $R_0(B_0)$ are our initial guesses for the iterative process.

The dominant eigenvector $\mathbf{q}^*$ in equation (19) is the infected population distribution. We use that alongside the factor ϕ^*, to fit our initial infected population distribution based on hospitalization data. Let $H_{T,obs}$ be the number of new hospitalizations observed on our data-set (SIVEP-Gripe) at a given date T, to find proper initial conditions, we need the total number of new hospitalizations given by the model to be equal to $H_{T,obs}$. For that, we'll need the terms $\sigma\gamma E$ in each of the H^{t+1}_{jkx}. Let each index d in the interval (n_l,n_h) correspond to an exposed class set of indexes, that is, $\phi^* q^*_d = E^0_{j,k,x}$ for all $n_l \le d \le n_h$ and (j,k,x). We have

$$\phi^* \sum_{d=n_l}^{n_h} q^*_d \sigma_d \gamma_d = H_{T,obs} \implies \phi^* = \frac{H_{T,obs}}{\sum_{d=n_l}^{n_h} q^*_d \sigma_d \gamma_d} \tag{21}$$

That way, our initial condition for infected individuals is given by $\phi^*\mathbf{q}^*$.

4 Parameterization

We consider that Omicron has a 50% reduced risk of hospitalization compared to Delta, whereas we use the data from Salje et al. [25] to obtain age-specific infection-hospitalization rate. Due to the short time interval, we consider that it is not possible to have reinfections by Omicron in Omicron-recovered individuals (i.e. $\rho_{oo}=0$). And since we do not have Delta circulating in this model, we assume that $\rho_{dd}, \rho_{od}=0$ also. Finally, we assume a Beta-distributed cross immunity given by Delta against Omicron infections (ρ_{do}) from Ferguson [13]. Tables S2 and S3 describe the basic epidemiological parameters. Figures S1, S2, S3 and S4 describe vaccine effectiveness parameters in forest plot format.

Parameter	Description	Value	Source
age dist	Estimated age distribution of Brazilian states as 2020	-	IBGE [19]
γ	Incubation period of the disease	$3\cdot2$ days	Gozzi et al. [16]
σ	Infection hospitalization rate	Table S3	Salje et al. [25]
α	Proportion of asymptomatic individuals	Table S3	[0-20)[26]<br>[20-120)[28]
ν	Time to recovery for asymptomatic/mild symptomatic	11 days	-
ν_s	Time to recovery/death for hospitalized individuals	Table S3	SIVEP-Gripe [21]
μ	Proportion of hospitalized individuals that die	Table S3	SIVEP-Gripe [21]

Table S2: Parameters used in the model with sources.

Age Group	σ	μ	α	$1-\alpha$	ν_s
0-4	0·0005	0·0712	0·6950	0·3050	9·2700
5-11	0·0005	0·0822	0·6950	0·3050	9·4464
12-17	0·0005	0·1104	0·6950	0·3050	9·9000
18-29	0·0022	0·1276	0·4788	0·5212	9·1032
30-39	0·0055	0·1552	0·4400	0·5600	9·4500
40-49	0·0070	0·2085	0·4400	0·5600	10·410
50-59	0·0145	0·2874	0·4400	0·5600	11·610
60-69	0·0290	0·4077	0·3100	0·6900	12·710
70-79	0·0465	0·5165	0·3100	0·6900	12·790
80+	0·1310	0·6162	0·3100	0·6900	11·426

Table S3: Age dependent parameters.

Study	VE (95% CI)	Product	Age	Used?
Assumed	0 (N/A)	AZD1222:D1	Assumed	Parameter
Barnard et al (pessimistic)	12.9 (N/A)	AZD1222:D1	NS	No
Barnard et al (optimistic)	23.7 (N/A)	AZD1222:D1	NS	No
Barnard et al (pessimistic)	18.7 (N/A)	BNT162b2:D1	NS	Sensitivity
Barnard et al (optimistic)	34.2 (N/A)	BNT162b2:D1	NS	No
Assumed	0 (N/A)	Coronavac:D1	18+	Parameter
Tseng et al	20 (8.9 − 29.8)	mRNA−1273:D1	18+	Parameter
Young Xu et al	25 (20 − 30)	Any mRNA:D2	65+	No
Ferguson et al	−39 (−50 − −30)	AZD1222:D2	Protocol	No
Spensley et al	−4 (−97 − 43)	AZD1222:D2	NS	Sensitivity
Willet et al	11.4 (−18.8 − 34.6)	AZD1222:D2	18+	Parameter
Barnard et al (pessimistic)	19 (N/A)	AZD1222:D2	NS	No
Barnard et al (optimistic)	34.7 (N/A)	AZD1222:D2	NS	No
Spensley et al	17 (−62 − 57)	BNT162b2:D2	NS	No
Hogan et al	19.1 (15.9 − 22.7)	BNT162b2:D2	16+	No
Barnard et al (pessimistic)	24.1 (N/A)	BNT162b2:D2	NS	No
Andeweg et al	25 (21 − 29)	BNT162b2:D2	18+	No
Willet et al	26 (13.9 − 36.4)	BNT162b2:D2	18+	No
Barnard et al (optimistic)	44.1 (N/A)	BNT162b2:D2	NS	No
Hansen et al	55.2 (23.5 − 73.5)	BNT162b2:D2	12+	No
NY Health Dept of Health	61 (N/A)	BNT162b2:D2	12 to 15	Parameter
NY Health Dept of Health	87 (N/A)	BNT162b2:D2	5 to 11	Parameter
Assumed	0 (N/A)	Coronavac:D2	18+	Parameter
Tseng et al	15.5 (12.2 − 18.7)	mRNA−1273:D2	18+	No
Young Xu et al	62 (59 − 65)	Any mRNA:D3	65+	No
Ferguson et al	55 (46 − 63)	AZD1222:D3	Protocol	No
Willet et al	43.2 (38.1 − 47.8)	BNT162b2:D3	18+	Sensitivity
Barnard et al (pessimistic)	44.1 (N/A)	BNT162b2:D3	NS	No
Abu−Raddad et al	47.7 (46 − 49.3)	BNT162b2:D3	Protocol	No
Hogan et al	48.4 (43.1 − 53.5)	BNT162b2:D3	16+	No
Spensley et al	66 (36 − 81)	BNT162b2:D3	NS	No
Accorsi et al	67.3 (65 − 69.4)	BNT162b2:D3	18+	No
Andeweg et al	68 (67 − 70)	BNT162b2:D3	18+	No
Barnard et al (optimistic)	79.2 (N/A)	BNT162b2:D3	NS	No
Tseng et al	63.6 (57.4 − 68.9)	mRNA−1273:D3	18+	No

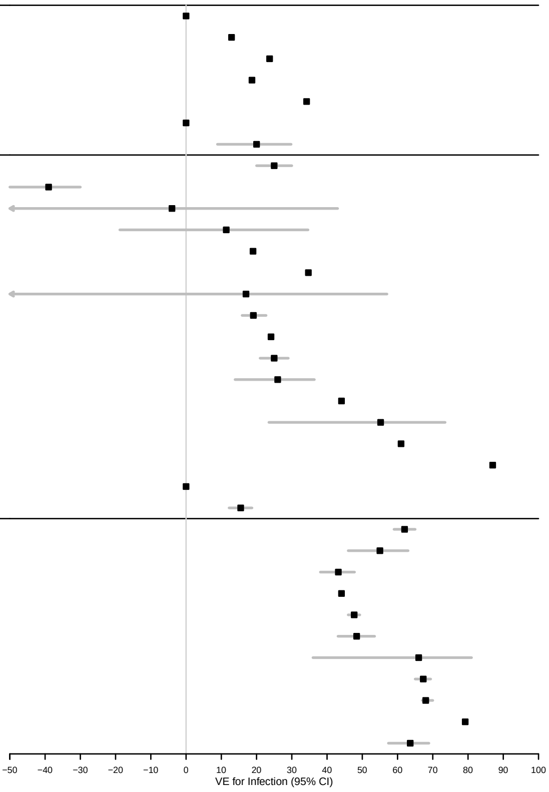

Figure S1: Forest plot of effectiveness studies regarding protection against COVID-19 infection. The studies are organized per dose and alphabetical order of vaccine name (See Product column). Used? column describes which studies were used in the main results (Parameter), sensitivity analysis (Sensitivity) or not used at all (No), but considered in the literature review. The studies are, in order of appearance: Barnard et al. [6], Tseng et al. [31], Young-Xu et al. [34], Ferguson [13], Spensley et al. [27], Willett et al. [33], Hogan et al. [18], Andeweg et al. [4], Hansen et al. [17], NY Dept of Health [22], Abu-Raddad et al. [1], Accorsi et al. [2].

Study	VE (95% CI)	Product	Age	Used?
Assumed*	26.1 (N/A)	AZD1222:D1	Assumed	Parameter
Barnard et al (pessimistic)	26.7 (N/A)	AZD1222:D1	NS	No
Barnard et al (optimistic)	35.7 (N/A)	AZD1222:D1	NS	No
Ferguson et al	2 (−7 − 11)	BNT162b2:D1	Protocol	No
Barnard et al (pessimistic)	18.7 (N/A)	BNT162b2:D1	NS	No
Chemaitelly et al	26.1 (5.8 − 42)	BNT162b2:D1	Protocol	Parameter
Andrews et al	34.2 (−3.5 − 58.1)	BNT162b2:D1	12+	No
Barnard et al (optimistic)	34.2 (N/A)	BNT162b2:D1	NS	No
Powell et al	42.1 (36.7 − 46.9)	BNT162b3:D1	12 to 15	Parameter
Powell et al	33 (18.6 − 44.9)	BNT162b4:D1	16 to 17	Parameter
Assumed	0 (N/A)	Coronavac:D1	18+	Parameter
Buchan et al	36 (24 − 45)	Any mRNA:D2	Protocol	No
Ferguson et al	−5 (−13 − 3)	AZD1222:D2	Protocol	No
Barnard et al (pessimistic)	23.3 (N/A)	AZD1222:D2	NS	No
Andrews et al	24.81 (1.17 − 42.79)	AZD1222:D2	12+	Sensitivity
Barnard et al (optimistic)	38.2 (N/A)	AZD1222:D2	NS	No
UKHSA	49.8 (40.7 − 57.5)	AZD1222:D2	Protocol	Parameter
Ferguson et al	−18 (−26 − −11)	BNT162b2:D2	Protocol	No
Barnard et al (pessimistic)	27.9 (N/A)	BNT162b2:D2	NS	No
Barnard et al (optimistic)	46.9 (N/A)	BNT162b2:D2	NS	No
Assumed**	48.5 (N/A)	BNT162b2:D2	5 to 11	Parameter
Glatman−Freedman et al	48.5 (24.3 − 65)	BNT162b2:D2	12 to 15	Parameter
Chemaitelly et al	61.9 (49.9 − 71.1)	BNT162b2:D2	Protocol	No
UKHSA	65.8 (64.4 − 67.2)	BNT162b2:D2	Protocol	Parameter
Andrews et al	88 (65.9 − 95.8)	BNT162b2:D2	12+	No
Powell et al	73 (66.4 − 78.3)	BNT162b3:D2	12 to 15	Parameter
Powell et al	71.3 (69.3 − 73.1)	BNT162b4:D2	16 to 17	Parameter
Assumed	20.25 (N/A)	Coronavac:D2	18+	Parameter
Buchan et al	60 (55 − 65)	Any mRNA:D3	Protocol	No
UKHSA	62.7 (62 − 63.4)	AZD1222:D3	Protocol	No
Andrews et al	71.4 (41.8 − 86)	AZD1222:D3	12+	Sensitivity
Chemaitelly et al	37.6 (28.8 − 45.4)	BNT162b2:D3	Protocol	No
Barnard et al (pessimistic)	46.9 (N/A)	BNT162b2:D3	NS	No
Abu−Raddad et al	50.1 (47.3 − 52.8)	BNT162b2:D3	Protocol	No
Andrews et al	75.5 (56.1 − 86.3)	BNT162b2:D3	12+	Parameter
Ferguson et al	77 (72 − 80)	BNT162b2:D3	Protocol	No
Barnard et al (optimistic)	80.3 (N/A)	BNT162b2:D3	NS	No

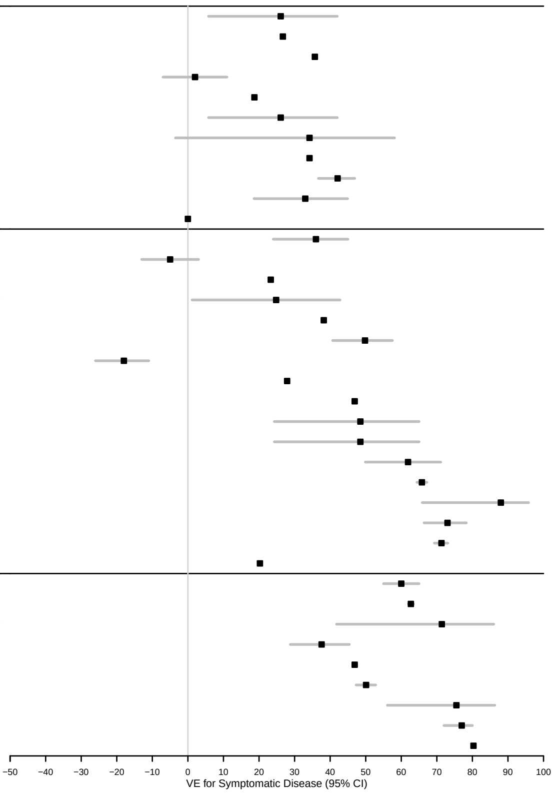

Figure S2: Forest plot of effectiveness studies regarding protection against COVID-19 symptomatic disease. The studies are organized per dose and alphabetical order of vaccine name (See Product column). Used? column describes which studies were used in the main results (Parameter), sensitivity analysis (Sensitivity) or not used at all (No), but considered in the literature review. The studies are, in order of appearance: Barnard et al. [6], Ferguson [13], Chemaitelly et al. [9], Andrews et al. [5], Powell et al. [23], Buchan et al. [7], UK Health Security Agency [32], Glatman-Freedman et al. [15],Abu-Raddad et al. [1].

Study	VE (95% CI)	Product	Age	Used?
Barnard et al (pessimistic)	49.7 (N/A)	AZD1222:D1	NS	Parameter
Barnard et al (optimistic)	66.3 (N/A)	AZD1222:D1	NS	No
Barnard et al (pessimistic)	59.6 (N/A)	BNT162b2:D1	NS	Parameter
Assumed***	59.6 (N/A)	BNT162b2:D1	5 to 18	Parameter
Barnard et al (optimistic)	76.7 (N/A)	BNT162b2:D1	NS	No
Assumed	0 (N/A)	Coronavac:D1	18+	Parameter
Ferdinands et al	55 (50 − 60)	Any mRNA:D2	12+	No
Lauring et al	65 (51 − 75)	Any mRNA:D2	18+	No
Thompson et al	81 (65 − 90)	Any mRNA:D2	12+	Parameter
Barnard et al (pessimistic)	60 (N/A)	AZD1222:D2	NS	Parameter
Barnard et al (optimistic)	77.1 (N/A)	AZD1222:D2	NS	No
Hogan et al	59.8 (54.3 − 65.1)	BNT162b2:D2	Protocol	Sensitivity
UKHSA	64 (62 − 65.9)	BNT162b2:D2	Protocol	Parameter
Assumed***	66.8 (N/A)	BNT162b2:D2	5 to 18	Parameter
Barnard et al (pessimistic)	66.8 (N/A)	BNT162b2:D2	NS	Parameter
Collie et al	69 (48 − 81)	BNT162b2:D2	NS	No
Tartof et al	70 (41 − 84)	BNT162b2:D2	12+	No
NY Health Dept of Health	76 (N/A)	BNT162b2:D2	5 to 11	Parameter
NY Health Dept of Health	81 (N/A)	BNT162b2:D2	12 to 15	Parameter
Barnard et al (optimistic)	83.7 (N/A)	BNT162b2:D2	NS	No
Assumed	40.65 (N/A)	Coronavac:D2	18+	Parameter
Tseng et al	74.8 (2.4 − 93.5)	mRNA−1273:D2	18+	No
Lauring et al	86 (77 − 91)	Any mRNA:D3	18+	No
Ferdinands et al	88 (86 − 90)	Any mRNA:D3	12+	No
Thompson et al	90 (80 − 94)	Any mRNA:D3	12+	Parameter
Spensley et al	60 (−163 − 90)	BNT162b2:D3	NS	No
UKHSA	68.7 (67.9 − 69.5)	BNT162b2:D3	Protocol	Parameter
Barnard et al (pessimistic)	83.7 (N/A)	BNT162b2:D3	NS	No
Hogan et al	85.5 (82.6 − 87.9)	BNT162b2:D3	Protocol	Sensitivity
Tartof et al	89 (84 − 92)	BNT162b2:D3	12+	No
Barnard et al (optimistic)	96.9 (N/A)	BNT162b2:D3	NS	No

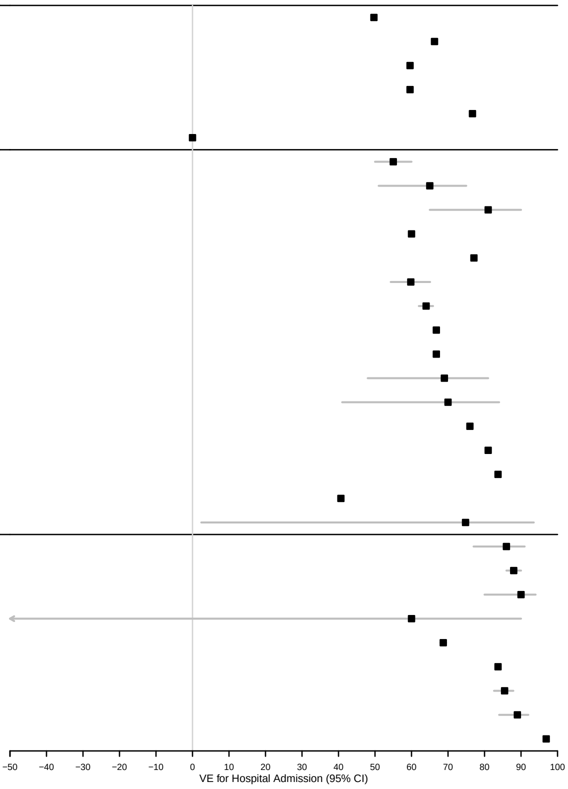

Figure S3: Forest plot of effectiveness studies regarding protection against COVID-19 hospital admission. The studies are organized per dose and alphabetical order of vaccine name (See Product column). Used? column describes which studies were used in the main results (Parameter), sensitivity analysis (Sensitivity) or not used at all (No), but considered in the literature review. The studies are, in order of appearance: Barnard et al. [6], Ferdinands et al. [12], Lauring et al. [20], Thompson et al. [30], Hogan et al. [18], UK Health Security Agency [32], Collie et al. [10], Tartof et al. [29], NY Dept of Health [22], Tseng et al. [31], Spensley et al. [27].

Study	VE (95% CI)	Product	Age	Used?
Barnard et al (pessimistic)	49.7 (N/A)	AZD1222:D1	NS	Parameter
Barnard et al (optimistic)	66.3 (N/A)	AZD1222:D1	NS	No
Barnard et al (pessimistic)	59.6 (N/A)	BNT162b2:D1	NS	Parameter
Barnard et al (optimistic)	76.7 (N/A)	BNT162b2:D1	NS	No
Assumed	0 (N/A)	Coronavac:D1	18+	Parameter
Young Xu et al	76 (62 − 85)	Any mRNA:D2	65+	Parameter
Barnard et al (pessimistic)	60 (N/A)	AZD1222:D2	NS	Parameter
Barnard et al (optimistic)	77.1 (N/A)	AZD1222:D2	NS	No
Barnard et al (pessimistic)	66.8 (N/A)	BNT162b2:D2	NS	No
Barnard et al (optimistic)	83.7 (N/A)	BNT162b2:D2	NS	No
Assumed	40.65 (N/A)	Coronavac:D2	18+	Parameter
Young Xu et al	96 (91 − 98)	Any mRNA:D3	65+	Parameter
Barnard et al (pessimistic)	83.7 (N/A)	BNT162b2:D3	NS	No
Barnard et al (optimistic)	96.9 (N/A)	BNT162b2:D3	NS	No

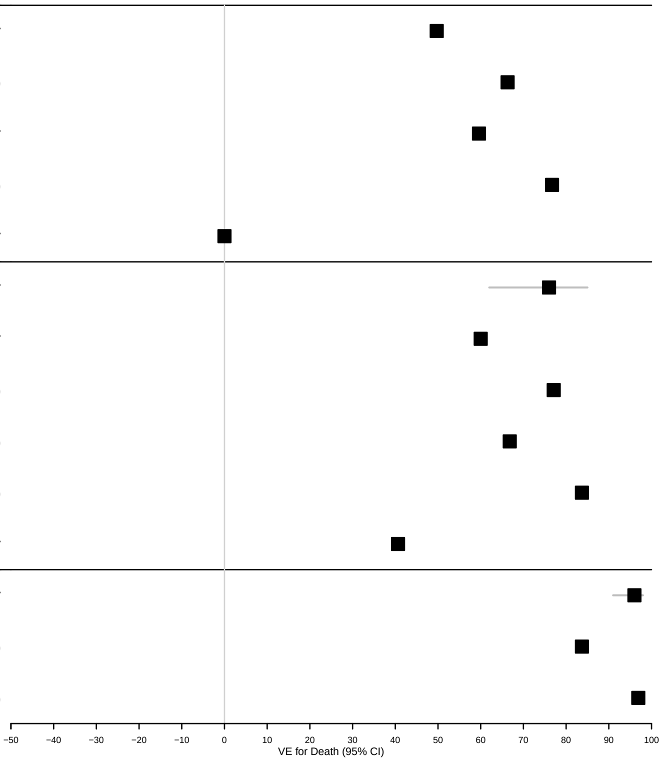

Figure S4: Forest plot of effectiveness studies regarding protection against COVID-19 death. The studies are organized per dose and alphabetical order of vaccine name (See Product column). Used? column describes which studies were used in the main results (Parameter), sensitivity analysis (Sensitivity) or not used at all (No), but considered in the literature review. The studies are, in order of appearance: Barnard et al. [6], Young-Xu et al. [34].

5 Additional results

In Table S4 we show the total number of events expected during the simulation.

Outcome	Scenario	Number of events [95% CI]
Cases	No vaccination	168·844 [163·428-174·243]
Cases	Current vaccination	166·713 [161·034-172·049]
Cases	Maximum vaccination	162·734 [156·949-168·268]
Hospitalizations	No vaccination	1·148 [1·096-1·206]
Hospitalizations	Current vaccination	1·138 [1·087-1·196]
Hospitalizations	Maximum vaccination	1·121 [1·071-1·178]
Deaths	No vaccination	0·214 [0·194-0·241]
Deaths	Current vaccination	0·213 [0·192-0·240]
Deaths	Maximum vaccination	0·210 [0·190-0·236]

Table S4: Total number of events, per outcome and vaccination scenario, in millions.

We do a sensitivity analysis of the results changing vaccine parameterization to other sources (see Figs S1, S2, S3 and S4). The number of deaths and hospitalizations avoided are described in Table S5. In Figures S5 and S6, we see the heterogeneity of the outcomes between states. Fig S7 describes a possible explanation for such heterogeneity.

Outcome	Age Group	Current Pace	Ideal Pace
Hospitalization	All	6 · 16 [5 · 35 – 7 · 17]	15 · 3 [13 · 09 – 18 · 44]
Death	All	1 · 54 [1 · 31 – 1 · 86]	4 · 31 [3 · 64 – 5 · 27]
Hospitalization	5-11	2 · 39 [2 · 25 – 2 · 55]	5 · 40 [5 · 07 – 5 · 74]
Death	5-11	0 · 18 [0 · 17 – 0 · 19]	0 · 41 [0 · 38 – 0 · 44]

Table S5: Number of outcome averted by age group and vaccination pace, in thousands, with 95% Confidence Interval.

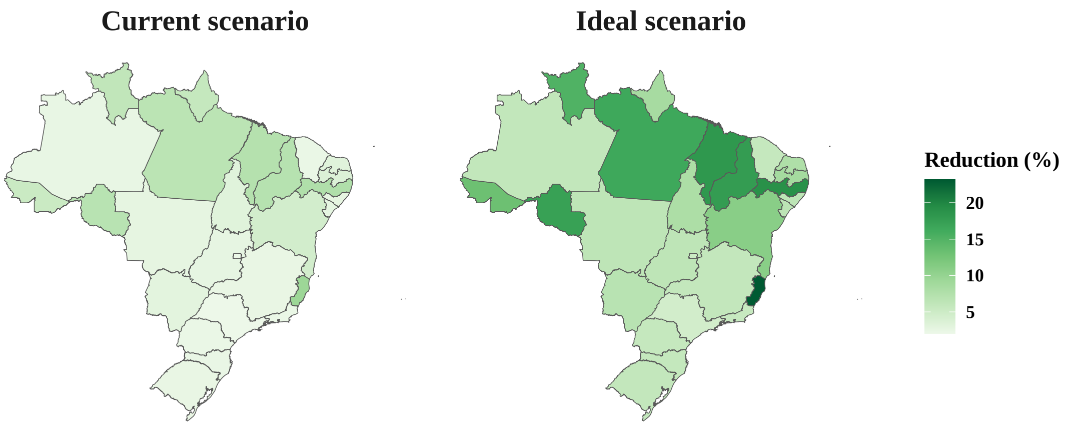

Figure S5: Mean number of percentage of hospitalizations reduced by vaccination in 5-11 age group, by state.

References

[1] Laith J. Abu-Raddad et al. "Effect of mRNA Vaccine Boosters against SARS-CoV-2 Omicron Infection in Qatar". In: *New England Journal of Medicine* (Mar. 2022). DOI: 10.1056/nejmoa2200797. URL: https://doi.org/10.1056%5C%2Fnejmoa2200797.

[2] Emma K. Accorsi et al. "Association Between 3 Doses of mRNA COVID-19 Vaccine and Symptomatic Infection Caused by the SARS-CoV-2 Omicron and Delta Variants". In: *JAMA* 327.7 (Feb. 2022), p. 639. DOI: 10.1001/jama.2022.0470. URL: https://doi.org/10.1001%5C%2Fjama.2022.0470.

[3] Linda JS Allen and P Van den Driessche. "The basic reproduction number in some discrete-time epidemic models". In: *Journal of difference equations and applications* 14.10-11 (2008), pp. 1127–1147.

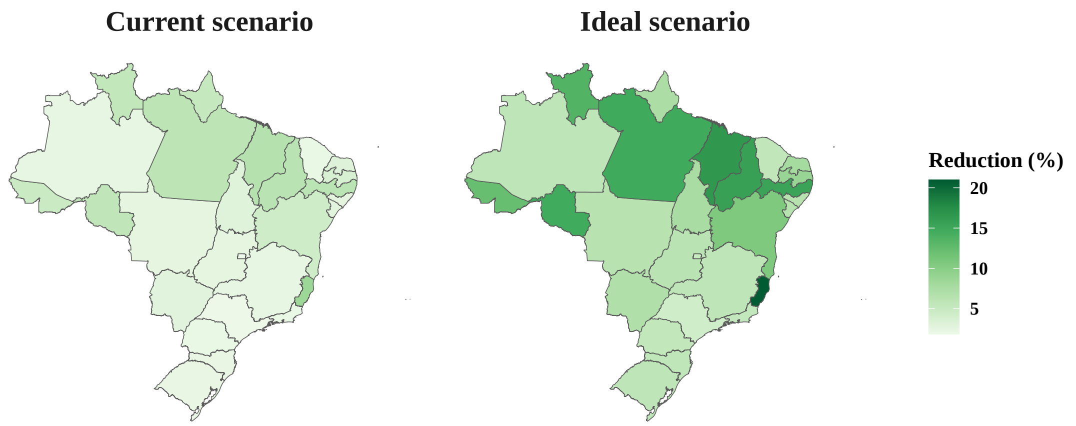

Figure S6: Mean number of percentage of deaths reduced by vaccination in 5-11 age group, by state.

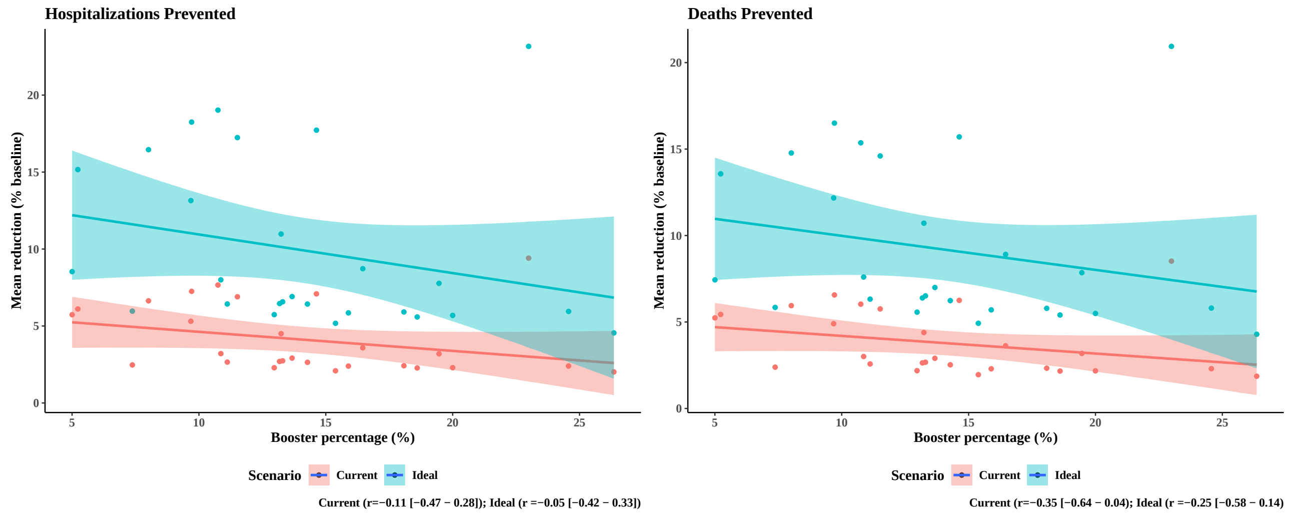

Figure S7: Linear regression of the mean number of percentage of hospitalizations reduced by vaccination in 5-11 age group, by state, versus coverage of booster doses in the whole population. Each point is a state.

[4] Stijn P. Andeweg et al. "Protection of COVID-19 vaccination and previous infection against Omicron BA.1 and Delta SARS-CoV-2 infections, the Netherlands, 22 November 2021- 19 January 2022". In: (Feb. 2022). DOI: 10.1101/2022.02.06.22270457. URL: https://doi.org/10.1101%5C%2F2022.02.06.22270457.

[5] Nick Andrews et al. "Covid-19 Vaccine Effectiveness against the Omicron (B.1.1.529) Variant". In: *New England Journal of Medicine* (Mar. 2022). DOI: 10.1056/nejmoa2119451. URL: https://doi.org/10.1056%5C%2Fnejmoa2119451.

[6] Rosanna C. Barnard et al. "Projected epidemiological consequences of the Omicron SARS-CoV-2 variant in England, December 2021 to April 2022". In: (Dec. 2021). DOI: 10.1101/2021.12.15.21267858. URL: https://doi.org/10.1101%5C%2F2021.12.15.21267858.

[7] Sarah A. Buchan et al. "Effectiveness of COVID-19 vaccines against Omicron or Delta symptomatic infection and severe outcomes". In: (Jan. 2022). DOI: 10.1101/2021.12.30.21268565. URL: https://doi.org/10.1101%5C%2F2021.12.30.21268565.

[8] Hal Caswell. *Matrix population models*. Vol. 1. Sinauer Sunderland, MA, 2000.

[9] Hiam Chemaitelly et al. "Duration of protection of BNT162b2 and mRNA-1273 COVID-19 vaccines against symptomatic SARS-CoV-2 Omicron infection in Qatar". In: (Feb. 2022). DOI: 10.1101/2022.02.07.22270568. URL: https://doi.org/10.1101%5C%2F2022.02.07.22270568.

[10] Shirley Collie et al. "Effectiveness of BNT162b2 Vaccine against Omicron Variant in South Africa". In: *New England Journal of Medicine* 386.5 (Feb. 2022), pp. 494–496. DOI: 10.1056/nejmc2119270. URL: https://doi.org/10.1056%5C%2Fnejmc2119270.

[11] Odo Diekmann et al. “On discrete time epidemic models in Kermack-McKendrick form”. In: (Mar. 2021). DOI: 10.1101/2021.03.26.21254385. URL: https://doi.org/10.1101/2021.03.26.21254385.

[12] Jill M. Ferdinands et al. “Waning 2-Dose and 3-Dose Effectiveness of mRNA Vaccines Against COVID-19–Associated Emergency Department and Urgent Care Encounters and Hospitalizations Among Adults During Periods of Delta and Omicron Variant Predominance — VISION Network, 10 States, August 2021–January 2022”. In: *MMWR. Morbidity and Mortality Weekly Report* 71.7 (Feb. 2022), pp. 255–263. DOI: 10.15585/mmwr.mm7107e2. URL: https://doi.org/10.15585%5C%2Fmmwr.mm7107e2.

[13] N Ferguson. *Report 49: Growth and immune escape of the Omicron SARS-CoV-2 variant of concern in England*. Tech. rep. 2021. DOI: 10.25561/93038. URL: http://spiral.imperial.ac.uk/handle/10044/1/93038.

[14] Leonardo Souto Ferreira et al. “Assessing the best time interval between doses in a two-dose vaccination regimen to reduce the number of deaths in an ongoing epidemic of SARS-CoV-2”. In: *PLOS Computational Biology* 18.3 (Mar. 2022). Ed. by Jennifer A. Flegg, e1009978. DOI: 10.1371/journal.pcbi.1009978. URL: https://doi.org/10.1371%2Fjournal.pcbi.1009978.

[15] Aharona Glatman-Freedman et al. “The BNT162b2 vaccine effectiveness against new COVID-19 cases and complications of breakthrough cases: A nation-wide retrospective longitudinal multiple cohort analysis using individualised data”. In: *eBioMedicine* 72 (Oct. 2021), p. 103574. DOI: 10.1016/j.ebiom.2021.103574. URL: https://doi.org/10.1016%5C%2Fj.ebiom.2021.103574.

[16] Nicolò Gozzi et al. “Preliminary modeling estimates of the relative transmissibility and immune escape of the Omicron SARS-CoV-2 variant of concern in South Africa”. In: (Jan. 2022). DOI: 10.1101/2022.01.04.22268721. URL: https://doi.org/10.1101%5C%2F2022.01.04.22268721.

[17] Christian Holm Hansen et al. “Vaccine effectiveness against SARS-CoV-2 infection with the Omicron or Delta variants following a two-dose or booster BNT162b2 or mRNA-1273 vaccination series: A Danish cohort study”. In: (Dec. 2021). DOI: 10.1101/2021.12.20.21267966. URL: https://doi.org/10.1101%5C%2F2021.12.20.21267966.

[18] A Hogan et al. *Report 48: The value of vaccine booster doses to mitigate the global impact of the Omicron SARS-CoV-2 variant*. Tech. rep. 2021. DOI: 10.25561/93034. URL: http://spiral.imperial.ac.uk/handle/10044/1/93034.

[19] Instituto Brasileiro de Geografia e Estatística. *Projeções da população*. 2021. URL: https://www.ibge.gov.br/estatisticas/sociais/populacao/9109-projecao-da-populacao.html (visited on 10/01/2021).

[20] Adam S Lauring et al. “Clinical severity of, and effectiveness of mRNA vaccines against, covid-19 from omicron, delta, and alpha SARS-CoV-2 variants in the United States: prospective observational study”. In: *BMJ* (Mar. 2022), e069761. DOI: 10.1136/bmj-2021-069761. URL: https://doi.org/10.1136%5C%2Fbmj-2021-069761.

[21] Ministério da Saúde. *SRAG 2021 - Banco de Dados de Síndrome Respiratória Aguda Grave - incluindo dados da COVID-19*. 2021. URL: https://opendatasus.saude.gov.br/dataset/bd-srag-2021 (visited on 07/28/2021).

[22] NY Dept of Health. *Pediatric COVID-19 update: January 7, 2022*. 2022. URL: https://health.ny.gov/press/releases/2022/docs/pediatric_covid-19_hospitalization_report.pdf (visited on 01/07/2022).

[23] Annabel A Powell et al. “Adolescent vaccination with BNT162b2 (Comirnaty, Pfizer-BioNTech) vaccine and effectiveness against COVID-19: national test-negative case-control study, England”. In: (Dec. 2021). DOI: 10.1101/2021.12.10.21267408. URL: https://doi.org/10.1101%5C%2F2021.12.10.21267408.

[24] Kiesha Prem et al. “Projecting contact matrices in 177 geographical regions: An update and comparison with empirical data for the COVID-19 era”. In: *PLOS Computational Biology* 17.7 (July 2021), pp. 1–19. DOI: 10.1371/journal.pcbi.1009098. URL: https://doi.org/10.1371/journal.pcbi.1009098.

[25] Henrik Salje et al. “Estimating the burden of SARS-CoV-2 in France”. In: *Science* 369.6500 (May 2020), pp. 208–211. DOI: 10.1126/science.abc3517. URL: https://doi.org/10.1126/science.abc3517.

[26] Secretaria Municipal de Saúde - Município de São Paulo. *Inquérito sorológico para Sars-Cov-2: Prevalência da infecção em escolares das redes públicas e privada da cidade de São Paulo*. 2021. URL: http://www.capital.sp.gov.br/arquivos/pdf/2021/coletiva_saude_14-01.pdf (visited on 01/31/2021).

[27] Katrina Spensley et al. “Comparison of vaccine effectiveness against the Omicron (B.1.1.529) variant in patients receiving haemodialysis”. In: (Jan. 2022). DOI: 10.1101/2022.01.25.22269804. URL: https://doi.org/10.1101%5C%2F2022.01.25.22269804.

[28] W. W. Sun et al. “Epidemiological characteristics of COVID-19 family clustering in Zhejiang Province”. In: *Chinese journal of preventive medicine* 54.6 (2020), pp. 625–629. ISSN: 02539624. DOI: 10.3760/cma.j.cn112150-20200227-00199.

[29] Sara Y. Tartof et al. “BNT162b2 (Pfizer–Biontech) mRNA COVID-19 Vaccine Against Omicron-Related Hospital and Emergency Department Admission in a Large US Health System: A Test-Negative Design”. In: *SSRN Electronic Journal* (2022). DOI: 10.2139/ssrn.4011905. URL: https://doi.org/10.2139%5C%2Fssrn.4011905.

[30] Mark G. Thompson et al. “Effectiveness of a Third Dose of mRNA Vaccines Against COVID-19–Associated Emergency Department and Urgent Care Encounters and Hospitalizations Among Adults During Periods of Delta and Omicron Variant Predominance — VISION Network, 10 States, August 2021–January 2022”. In: *MMWR. Morbidity and Mortality Weekly Report* 71.4 (Jan. 2022), pp. 139–145. DOI: 10.15585/mmwr.mm7104e3. URL: https://doi.org/10.15585%5C%2Fmmwr.mm7104e3.

[31] Hung Fu Tseng et al. “Effectiveness of mRNA-1273 against SARS-CoV-2 Omicron and Delta variants”. In: *Nature Medicine* (Feb. 2022). DOI: 10.1038/s41591-022-01753-y. URL: https://doi.org/10.1038%5C%2Fs41591-022-01753-y.

[32] UK Health Security Agency. *COVID-19 vaccine surveillance report, Week 5, 3 February 2022*. 2022. URL: https://assets.publishing.service.gov.uk/government/uploads/system/uploads/attachment_data/file/1052353/Vaccine_surveillance_report_-_week_5.pdf (visited on 02/03/2022).

[33] Brian J. Willett et al. “The hyper-transmissible SARS-CoV-2 Omicron variant exhibits significant antigenic change, vaccine escape and a switch in cell entry mechanism”. In: (Jan. 2022). DOI: 10.1101/2022.01.03.21268111. URL: https://doi.org/10.1101%5C%2F2022.01.03.21268111.

[34] Yinong Young-Xu et al. “Effectiveness of mRNA COVID-19 Booster Vaccines against Omicron and Delta Variants among US Veterans”. In: (Jan. 2022). DOI: 10.1101/2022.01.15.22269360. URL: https://doi.org/10.1101%5C%2F2022.01.15.22269360.
